# The genome sequence of Schreibers’s Long-fingered Bat, *Miniopterus schreibersii* (Kuhl, 1817) (Chiroptera: Miniopteridae)

**DOI:** 10.12688/wellcomeopenres.26286.1

**Published:** 2026-04-22

**Authors:** Ine Alvarez van Tussenbroek, Sonja C. Vernes, Haris Nicolaou, Emma C. Teeling, Meike Mai, Myrtani Pieri, Bat1K Consortium, Wellcome Sanger Institute Tree of Life Management, Samples and Laboratory team, Wellcome Sanger Institute Scientific Operations: Sequencing Operations, Wellcome Sanger Institute Tree of Life Core Informatics team, Tree of Life Core Informatics collective, Darwin Tree of Life Consortium

**Affiliations:** 1 University of St Andrews School of Biology, St Andrews, Scotland, UK; 2 Department of Forests, Ministry of Agriculture, Rural Development and Environment, Nicosia, Cyprus; 3 School of Biology and Environmental Science, University College Dublin, Dublin, Ireland; 4 Tree of Life Programme, Wellcome Sanger Institute, Hinxton, England, UK; 5 School of Life and Health Sciences, University of Nicosia, Nicosia, Nicosia, Cyprus

**Keywords:** Miniopterus schreibersii; Schreibers’s Long-fingered Bat; genome sequence; chromosomal; Chiroptera

## Abstract

We present a genome assembly from an individual male *Miniopterus schreibersii* (Schreibers’s Long-fingered Bat; Chordata; Mammalia; Chiroptera; Miniopteridae). The assembly contains two haplotypes with total lengths of 1 850.57 megabases and 1 699.63 megabases. Most of haplotype 1 (97.59%) is scaffolded into 24 chromosomal pseudomolecules, including the X and Y sex chromosomes. Haplotype 2 was assembled to scaffold level. The mitochondrial genome has also been assembled, with a length of 16.63 kilobases. Gene annotation of this assembly on Ensembl identified 18 289 protein-coding genes. This assembly was generated as part of the Darwin Tree of Life project, which produces reference genomes for eukaryotic species found in Britain and Ireland.

## Species taxonomy

Eukaryota; Opisthokonta; Metazoa; Eumetazoa; Bilateria; Deuterostomia; Chordata; Craniata; Vertebrata; Gnathostomata; Teleostomi; Euteleostomi; Sarcopterygii; Dipnotetrapodomorpha; Tetrapoda; Amniota; Mammalia; Theria; Eutheria; Boreoeutheria; Laurasiatheria; Chiroptera; Yangochiroptera; Miniopteridae; Miniopterus; *Miniopterus schreibersii* (Kuhl, 1817) (NCBI:txid9433).

## Background

The Schreiber’s bent-winged bat, *Miniopterus schreibersii* is a medium-sized bat with long, narrow wings with a pronounced “bend” at the third finger, a short domed head, and soft brownish-grey fur ( Kuhl, 1819). Its common name, ‘common bent-wing bat’, derives from the curvature of the wing, and it is also referred as ‘Schreiber’s long-fingered bat’ or ‘Schreiber’s bat’. Identification is primarily based on its characteristic wing shape and cranial features, although cryptic species within the complex can only be reliably distinguished using genetic and cranio-dental analyses, as subtle external differences are insufficient for field identification ( Puechmaille *et al.*, 2014).

The species has a broad distribution extending across southern Europe, North Africa, the Middle East, the Caucasus, Central and East Asia, and into Australasia. GBIF.org records confirm this extensive range, with populations documented from the Iberian Peninsula through the Balkans and Anatolia to regions of the Palaearctic and Ethiopian biogeographic zones ( Appleton *et al.*, 2004; Bilgin *et al.*, 2016; Puechmaille *et al.*, 2014). Phylogeographic studies indicate that *M. schreibersii* recolonized Europe from glacial refugia in the eastern Mediterranean following the last glacial maximum, while additional cryptic lineages persist in North Africa ( Bilgin *et al.*, 2016; Puechmaille *et al.*, 2014). This evolutionary history reflects both the species’ high dispersal capability and its ability to adapt to diverse climatic and ecological conditions.

Typical habitats include caves, tunnels, and abandoned mines, where large colonies form dense aggregations. The species is highly gregarious and migratory, with some individuals undertaking long-distance seasonal movements, particularly across eastern and southeastern Europe ( Wright *et al.*, 2020). Maternity colonies form in spring, consisting of females that give birth to a single pup annually, exhibiting strong philopatry to natal roosts ( Appleton *et al.*, 2004; Gürün *et al.*, 2019; Wright *et al.*, 2020).

*Miniopterus schreibersii* displays remarkable biological adaptations, including exceptional flight performance suited to long-distance migration, high gene flow among populations, and a complex social structure, characterised by spatial separation between mating and breeding sites ( Dufresnes *et al.*, 2023; Wright *et al.*, 2020). The genus *Miniopterus* is also notable for its cryptic speciation and was recently reclassified into its own family, Miniopteridae, based on molecular and morphological evidence ( Demos *et al.*, 2023; Miller-Butterworth *et al.*, 2007).

The species is listed as Near Threatened on the IUCN Red List due to population declines and habitat fragmentation, particularly in Europe ( Dufresnes *et al.*, 2023; Wood *et al.*, 2011). Conservation efforts are increasingly informed by molecular and tracking studies, which reveal population connectivity and migratory behavior across the continent.

High-resolution genomic and phylogenomic analyses, including RAD-seq and ultraconserved element (UCE) studies, have advanced understanding of population structure, cryptic diversity, and evolutionary history ( Demos *et al.*, 2023; Dufresnes *et al.*, 2023). The completed genome sequence provides a powerful resource for research into population genetics, disease ecology – given its role as a potential reservoir for zoonotic viruses – and conservation management. It further supports the development of tools for accurate species identification, long-term monitoring, and investigation of adaptive traits underlying flight, migration, and longevity ( Demos *et al.*, 2023; Dufresnes *et al.*, 2023).

**Figure 1.**
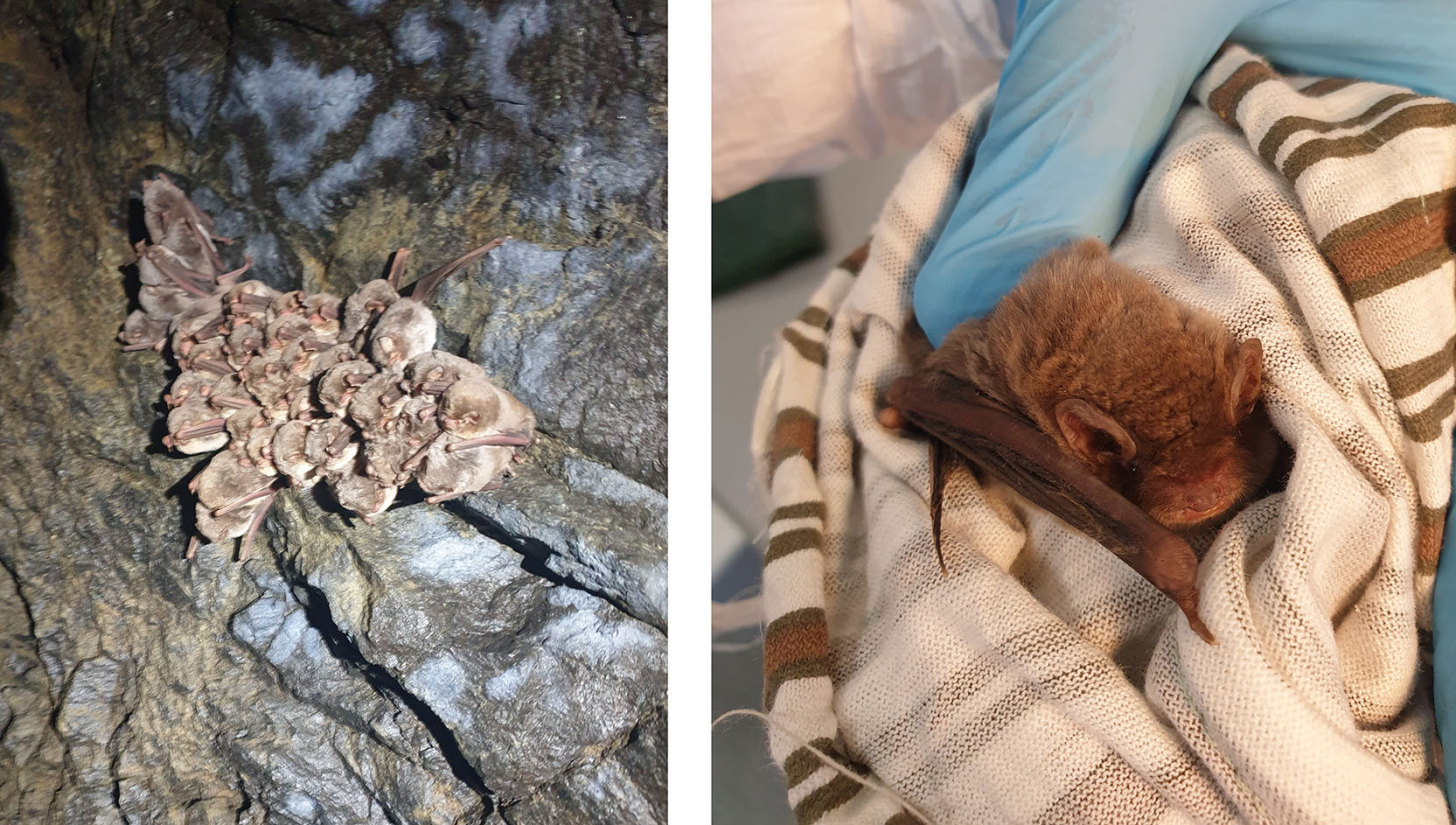
Common bent-wing bat, *Miniopterus schreibersii.* Individuals of *M. schreibersii.* (A-B) The specimen was collected from a group roosting in a cave in Cyprus. (Photos taken by Ine Alvarez van Tussenbroek).

Another scaffold-level genome assembly for this species is available (GCA_004026525.1; submitted by the Broad Institute). Here we present a chromosome-level genome sequence for *Miniopterus schreibersii.* The sequence is based on tissue obtained from a male collected in an abandoned mine in the Troodos mountains, Cyprus ( Figure 1). This species occupies a range of elevations depending on the season, occurring at low altitudes near the sea level (around 50 m) and at higher elevations up to about 1305 m above sea level ( Benda *et al.*, 2007). This sampling is part of the Bat1K Project ( Teeling *et al.*, 2018) and the Darwin Tree of Life Project (DToL) ( Darwin Tree of Life Project Consortium, 2022). The Bat1K Project is a collaborative effort to sequence all extant bat species, and DToL aims to sequence all named eukaryotic species in the Atlantic Archipelago of Britain and Ireland.

## Methods

### Sample acquisition

The specimen used for genome sequencing was an adult male *Miniopterus schreibersii* (specimen ID SAN00002494, ToLID mMinSch1; Figure 1). Groups of *M. schreibersii* consisting of 2 to 10 individuals were observed roosting on the ceiling of an abandoned mine in the Troodos mountains, Cyprus (latitude 35.1464, longitude 33.4098) on 2022-12-05. Sites of this species in Cyprus have been previously documented ( Benda *et al.*, 2007). One male individual was collected from these groups. The specimen was approximately 5.5 cm from head to bottom (excluding the tail). It was transported to a laboratory in Nicosia, where it was euthanised, dissected and multiple organs were extracted. Samples were placed in Eppendorf tubes and snap frozen in liquid nitrogen, and stored at −80 °C until it was shipped for sequencing.

### Nucleic acid extraction

Protocols for high molecular weight (HMW) DNA extraction developed at the Wellcome Sanger Institute (WSI) Tree of Life Core Laboratory are available on protocols.io ( Howard *et al.*, 2025). The mMinSch1 sample was weighed and triaged to determine the appropriate extraction protocol. Heart tissue was homogenised by powermashing using a PowerMasher II tissue disruptor. HMW DNA was extracted using the Automated MagAttract v2 protocol. DNA was sheared into an average fragment size of 12–20 kb following the Megaruptor®3 for LI PacBio protocol. Sheared DNA was purified by automated SPRI (solid-phase reversible immobilisation).

The concentration of the sheared and purified DNA was assessed using a Nanodrop spectrophotometer and Qubit Fluorometer using the Qubit dsDNA High Sensitivity Assay kit. Fragment size distribution was evaluated by running the sample on the FemtoPulse system. For this sample, the final post-shearing DNA had a Qubit concentration of 22.0 ng/μL and a yield of 2 860.00 ng.

RNA was extracted from brain tissue of mMinSch1 in the Tree of Life Laboratory at the WSI using the RNA Extraction: Automated MagMax™ *mir*Vana protocol. The RNA concentration was assessed using a Nanodrop spectrophotometer and a Qubit Fluorometer using the Qubit RNA Broad-Range Assay kit. Analysis of the integrity of the RNA was done using the Agilent RNA 6000 Pico Kit and Eukaryotic Total RNA assay.

### PacBio HiFi library preparation and sequencing

Library preparation and sequencing were performed at the WSI Scientific Operations core. Libraries were prepared using the SMRTbell Prep Kit 3.0 (Pacific Biosciences, California, USA), following the manufacturer’s instructions. The kit includes reagents for end repair/A-tailing, adapter ligation, post-ligation SMRTbell bead clean-up, and nuclease treatment. Size selection and clean-up were performed using diluted AMPure PB beads (Pacific Biosciences). DNA concentration was quantified using a Qubit Fluorometer v4.0 (ThermoFisher Scientific) and the Qubit 1X dsDNA HS assay kit. Final library fragment size was assessed with the Agilent Femto Pulse Automated Pulsed Field CE Instrument (Agilent Technologies) using the gDNA 55 kb BAC analysis kit.

The sample was sequenced on a Revio instrument (Pacific Biosciences). The prepared library was normalised to 2 nM, and 15 μL was used for making complexes. Primers were annealed and polymerases bound to generate circularised complexes, following the manufacturer’s instructions. Complexes were purified using 1.2X SMRTbell beads, then diluted to the Revio loading concentration (200–300 pM) and spiked with a Revio sequencing internal control. The sample was sequenced on a Revio 25 M SMRT cell. The SMRT Link software (Pacific Biosciences), a web-based workflow manager, was used to configure and monitor the run and to carry out primary and secondary data analysis.

### Hi-C

***Sample preparation and crosslinking***

The Hi-C sample was prepared from 20–50 mg of frozen heart tissue from the mMinSch1 sample using the Arima-HiC v2 kit (Arima Genomics). Following the manufacturer’s instructions, tissue was fixed and DNA crosslinked using TC buffer to a final formaldehyde concentration of 2%. The tissue was homogenised using the Diagnocine Power Masher-II. Crosslinked DNA was digested with a restriction enzyme master mix, biotinylated, and ligated. Clean-up was performed with SPRISelect beads before library preparation. DNA concentration was measured with the Qubit Fluorometer (Thermo Fisher Scientific) and Qubit HS Assay Kit. The biotinylation percentage was estimated using the Arima-HiC v2 QC beads.

***Hi-C library preparation and sequencing***

Biotinylated DNA constructs were fragmented using a Covaris E220 sonicator and size selected to 400–600 bp using SPRISelect beads. DNA was enriched with Arima-HiC v2 kit Enrichment beads. End repair, A-tailing, and adapter ligation were carried out with the NEBNext Ultra II DNA Library Prep Kit (New England Biolabs), following a modified protocol where library preparation occurs while DNA remains bound to the Enrichment beads. Library amplification was performed using KAPA HiFi HotStart mix and a custom Unique Dual Index (UDI) barcode set (Integrated DNA Technologies). Depending on sample concentration and biotinylation percentage determined at the crosslinking stage, libraries were amplified with 10–16 PCR cycles. Post-PCR clean-up was performed with SPRISelect beads. Libraries were quantified using the AccuClear Ultra High Sensitivity dsDNA Standards Assay Kit (Biotium) and a FLUOstar Omega plate reader (BMG Labtech).

Prior to sequencing, libraries were normalised to 10 ng/μL. Normalised libraries were quantified again to create equimolar and/or weighted 2.8 nM pools. Pool concentrations were checked using the Agilent 4200 TapeStation (Agilent) with High Sensitivity D500 reagents before sequencing. Sequencing was performed using paired-end 150 bp reads on the Illumina NovaSeq X.

### RNA library preparation and sequencing

Libraries were prepared using the NEBNext ^®^ Ultra™ II Directional RNA Library Prep Kit for Illumina (New England Biolabs), following the manufacturer’s instructions. Poly(A) mRNA in the total RNA solution was isolated using oligo (dT) beads, converted to cDNA, and uniquely indexed; 14 PCR cycles were performed. Libraries were size-selected to produce fragments between 100–300 bp. Libraries were quantified, normalised, pooled to a final concentration of 2.8 nM, and diluted to 150 pM for loading. Sequencing was carried out on the Illumina NovaSeq X, generating paired-end reads.

### Genome assembly

Prior to assembly of the PacBio HiFi reads, a database of *k*-mer counts ( *k* = 31) was generated from the filtered reads using FastK. GenomeScope2 ( Ranallo-Benavidez *et al.*, 2020) was used to analyse the *k*-mer frequency distributions, providing estimates of genome size, heterozygosity, and repeat content.

The HiFi reads were assembled using Hifiasm in Hi-C phasing mode ( Cheng *et al.*, 2021, 2022), producing two haplotypes. Hi-C reads ( Rao *et al.*, 2014) were mapped to the primary contigs using bwa-mem2 ( Vasimuddin *et al.*, 2019). Contigs were further scaffolded with Hi-C data in YaHS ( Zhou *et al.*, 2023), using the --break option for handling potential misassemblies. The scaffolded assemblies were evaluated using Gfastats ( Formenti *et al.*, 2022), BUSCO ( Manni *et al.*, 2021) and MerquryFK ( Rhie *et al.*, 2020).

The mitochondrial genome was assembled using MitoHiFi ( Uliano-Silva *et al.*, 2023).

### Assembly curation

The assembly was decontaminated using the Assembly Screen for Cobionts and Contaminants ( ASCC) pipeline. TreeVal was used to generate the flat files and maps for use in curation. Manual curation was conducted primarily in PretextView and HiGlass ( Kerpedjiev *et al.*, 2018). Scaffolds were visually inspected and corrected as described by Howe *et al*. (2021). Manual corrections included seven breaks and 55 joins. This reduced the scaffold count by 14.3%, increased the scaffold N50 by 3.4%, and increased the total assembly length by 0.5%. The curation process is described at https://gitlab.com/wtsi-grit/rapid-curation . PretextSnapshot was used to generate a Hi-C contact map of the final assembly.

### Assembly quality assessment

The MerquryFK tool ( Rhie *et al.*, 2020) was run in a Singularity container ( Kurtzer *et al.*, 2017) to evaluate *k*-mer completeness and assembly quality for both haplotypes using the *k*-mer databases ( *k* = 31) computed prior to genome assembly. The analysis outputs included assembly QV scores and completeness statistics.

The genome was analysed using the BlobToolKit pipeline, a Nextflow implementation of the earlier Snakemake version ( Challis *et al.*, 2020). The pipeline aligns PacBio reads using minimap2 ( Li, 2018) and SAMtools ( Danecek *et al.*, 2021) to generate coverage tracks. It runs BUSCO ( Manni *et al.*, 2021) using lineages identified from the NCBI Taxonomy ( Schoch *et al.*, 2020). For the three domain-level lineages, BUSCO genes are aligned to the UniProt Reference Proteomes database ( Bateman *et al.*, 2023) using DIAMOND blastp ( Buchfink *et al.*, 2021). The genome is divided into chunks based on the density of BUSCO genes from the closest taxonomic lineage, and each chunk is aligned to the UniProt Reference Proteomes database with DIAMOND blastx. Sequences without hits are chunked using seqtk and aligned to the NT database with blastn ( Altschul *et al.*, 1990). The BlobToolKit suite consolidates all outputs into a blobdir for visualisation. The BlobToolKit pipeline was developed using nf-core tooling ( Ewels *et al.*, 2020) and MultiQC ( Ewels *et al.*, 2016), with containerisation through Docker ( Merkel, 2014) and Singularity ( Kurtzer *et al.*, 2017).

## Genome sequence report

### Sequence data

**Figure 2.**
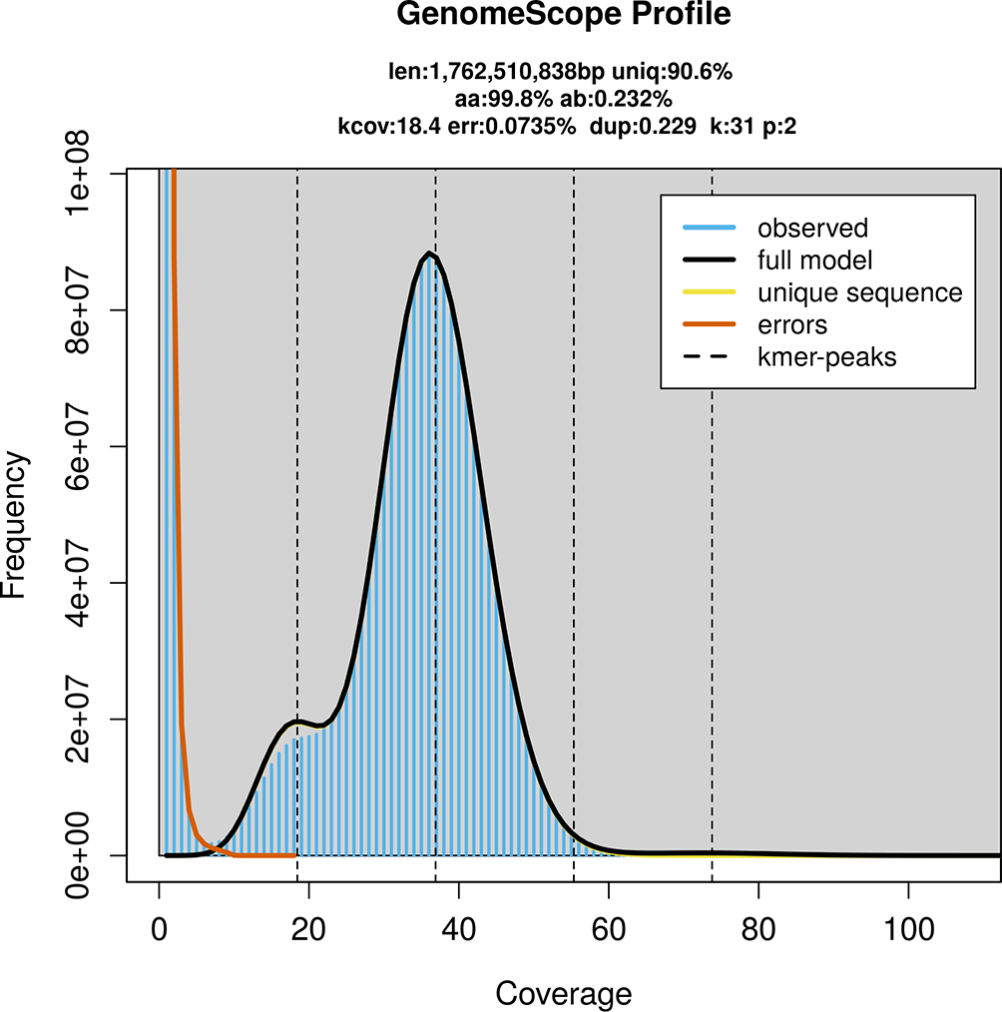
Frequency distribution of *k*-mers generated using GenomeScope2. The plot shows observed and modelled *k*-mer spectra, providing estimates of genome size, heterozygosity, and repeat content based on unassembled sequencing reads.

PacBio sequencing of the *Miniopterus schreibersii* specimen generated 66.71 Gb (gigabases) from 6.43 million reads, which were used to assemble the genome. GenomeScope2.0 analysis estimated the haploid genome size at 1 762.51 Mb, with a heterozygosity of 0.23% and repeat content of 9.47% ( Figure 2). These estimates guided expectations for the assembly. Based on the estimated genome size, the sequencing data provided approximately 37× coverage. Hi-C sequencing produced 204.66 Gb from 1 355.39 million reads, which were used to scaffold the assembly. RNA sequencing data were also generated and are available in public sequence repositories. Table 1 summarises the specimen and sequencing details.

**Table 1.** Specimen and sequencing data for BioProject PRJEB74978.

Platform	PacBio HiFi	Hi-C	RNA-seq
**ToLID**	mMinSch1	mMinSch1	mMinSch1
**Specimen ID**	SAN00002494	SAN00002494	SAN00002494
**BioSample (source individual)**	SAMEA113980738	SAMEA113980738	SAMEA113980738
**BioSample (tissue)**	SAMEA113980757	SAMEA113980757	SAMEA113980764
**Tissue**	heart	heart	brain
**Instrument**	Revio	Illumina NovaSeq X	Illumina NovaSeq X
**Run accessions**	ERR12921321	ERR12945475	ERR13493944
**Read count total**	6.43 million	1 355.39 million	89.43 million
**Base count total**	66.71 Gb	204.66 Gb	13.50 Gb

### Assembly statistics

The genome was assembled into two haplotypes using Hi-C phasing. Haplotype 1 was curated to chromosome level, while haplotype 2 was assembled to scaffold level. The final assembly has a total length of 1 850.57 Mb in 185 scaffolds, with 140 gaps, and a scaffold N50 of 87.8 Mb ( Table 2).

**Table 2.** Genome assembly statistics.

**Assembly name**	mMinSch1.hap1.2	mMinSch1.hap2.2
**Assembly accession**	GCA_964146895.2	GCA_964145185.2
**Assembly level**	chromosome	scaffold
**Span (Mb)**	1 850.57	1 699.63
**Number of chromosomes**	24	scaffold-level
**Number of contigs**	325	254
**Contig N50**	28.01 Mb	31.77 Mb
**Number of scaffolds**	185	168
**Scaffold N50**	87.8 Mb	87.05 Mb
**Longest scaffold length (Mb)**	202.31	-
**Sex chromosomes**	X and Y	-
**Organelles**	Mitochondrion: 16.63 kb	-

**Figure 3.**
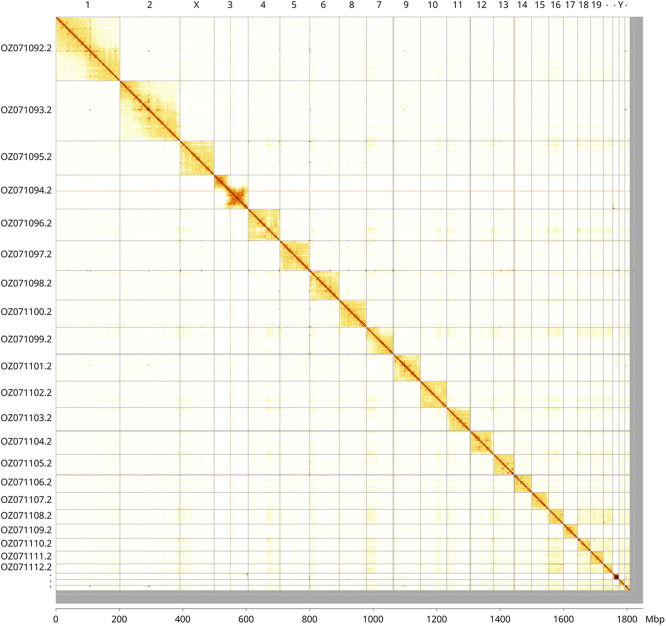
Hi-C contact map of the *Miniopterus schreibersii* genome assembly. Assembled chromosomes are shown in order of size and labelled along the axes, with a megabase scale shown below. The plot was generated using PretextSnapshot.

Most of the haplotype 1 assembly sequence (97.59%) was assigned to 24 chromosomal-level scaffolds, representing 22 autosomes and the X and Y sex chromosomes. These chromosome-level scaffolds, confirmed by Hi-C data, are named according to synteny ( Figure 3; Table 3). The order and orientation of contigs along the Y chromosome are uncertain in the region 6.6–15.5 Mb.

**Table 3.** Chromosomal pseudomolecules in the haplotype 1 genome assembly of *Miniopterus schreibersii* mMinSch1.

INSDC accession	Molecule	Length (Mb)	GC%
OZ071092.2	1	202.31	41
OZ071093.2	2	187.81	40
OZ071094.2	3	105.36	42
OZ071096.2	4	102.19	44
OZ071097.2	5	92.84	40.50
OZ071098.2	6	91.04	41
OZ071099.2	7	85.52	41
OZ071100.2	8	87.80	44
OZ071101.2	9	84.58	40.50
OZ071102.2	10	83.37	43.50
OZ071103.2	11	74.81	43.50
OZ071104.2	12	74.20	43.50
OZ071105.2	13	63.12	43.50
OZ071106.2	14	56.44	44
OZ071107.2	15	53.95	44
OZ071108.2	16	46.13	47
OZ071109.2	17	44.60	43
OZ071110.2	18	40.65	48
OZ071111.2	19	40.17	45.50
OZ071112.2	20	28.13	48
OZ071114.2	21	19.08	46.50
OZ071115.2	22	16.11	48
OZ071095.2	X	106.82	39
OZ071113.2	Y	18.87	45

The mitochondrial genome was also assembled (length 16.63 kb, OZ071116.2). This sequence is included as a contig in the multifasta file of the genome submission and as a standalone record.

### Assembly quality metrics

**Figure 4.**
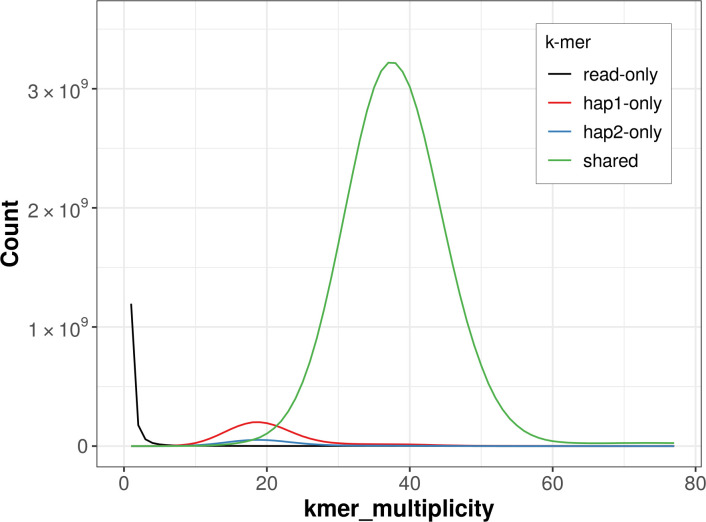
Evaluation of *k*-mer completeness using MerquryFK. This plot illustrates the recovery of *k*-mers from the original read data in the final assemblies. The horizontal axis represents *k*-mer multiplicity, and the vertical axis shows the number of *k*-mers. The black curve represents *k*-mers that appear in the reads but are not assembled. The green curve corresponds to *k*-mers shared by both haplotypes, and the red and blue curves show *k*-mers found only in one of the haplotypes.

For haplotype 1, the estimated QV is 61.8, and for haplotype 2, 61.7. When the two haplotypes are combined, the assembly achieves an estimated QV of 61.8. The *k*-mer completeness is 97.74% for haplotype 1, 91.88% for haplotype 2, and 99.79% for the combined haplotypes ( Figure 4).

**Figure 5.**
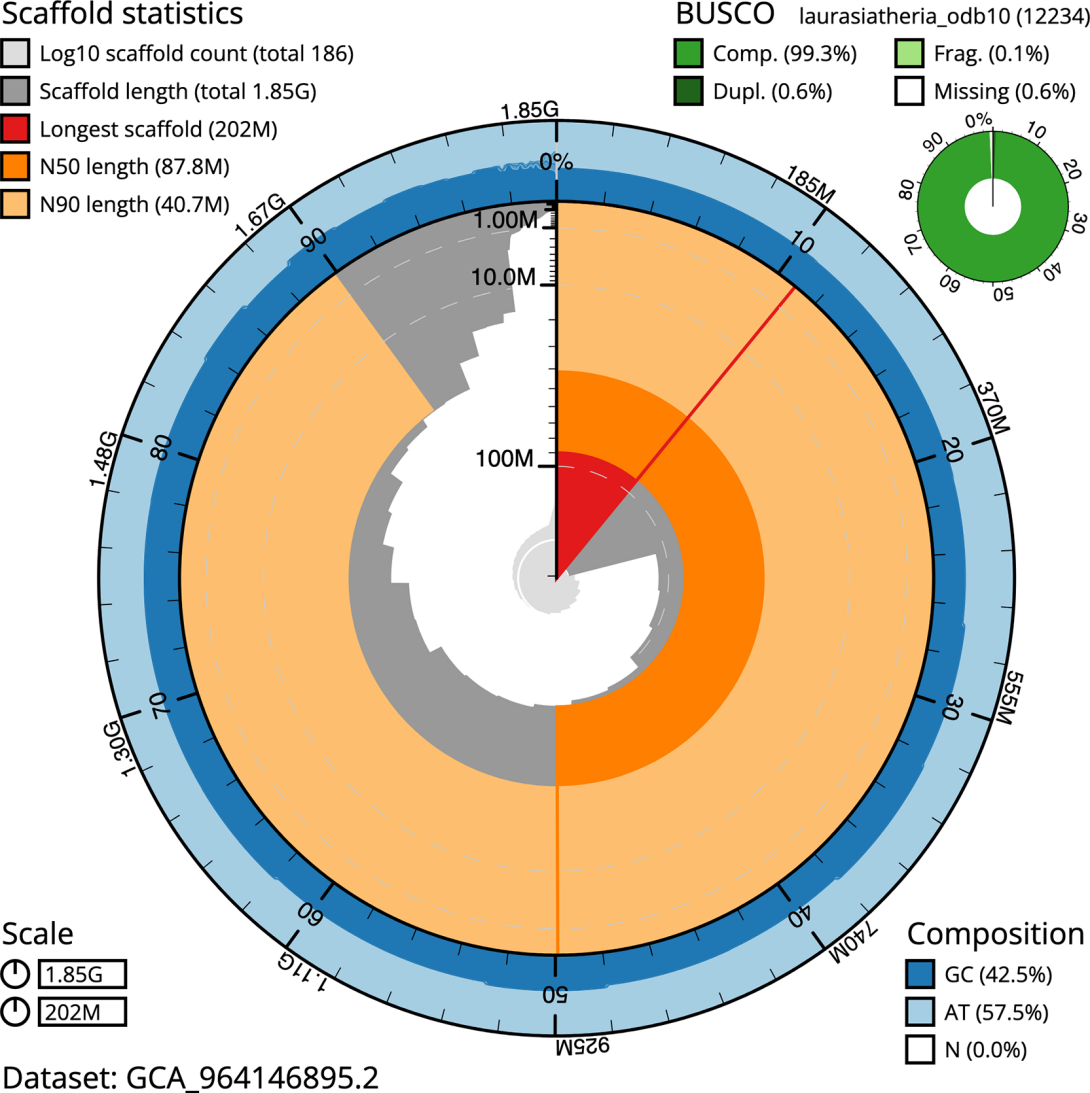
Assembly metrics for mMinSch1.hap1.2. The BlobToolKit snail plot provides an overview of assembly metrics and BUSCO gene completeness. The circumference represents the length of the whole genome sequence, and the main plot is divided into 1 000 bins around the circumference. The outermost blue tracks display the distribution of GC, AT, and N percentages across the bins. Scaffolds are arranged clockwise from longest to shortest and are depicted in dark grey. The longest scaffold is indicated by the red arc, and the deeper orange and pale orange arcs represent the N50 and N90 lengths. A light grey spiral at the centre shows the cumulative scaffold count on a logarithmic scale. A summary of complete, fragmented, duplicated, and missing BUSCO genes in the set is presented at the top right. An interactive version of this figure can be accessed on the BlobToolKit viewer.

**Figure 6.**
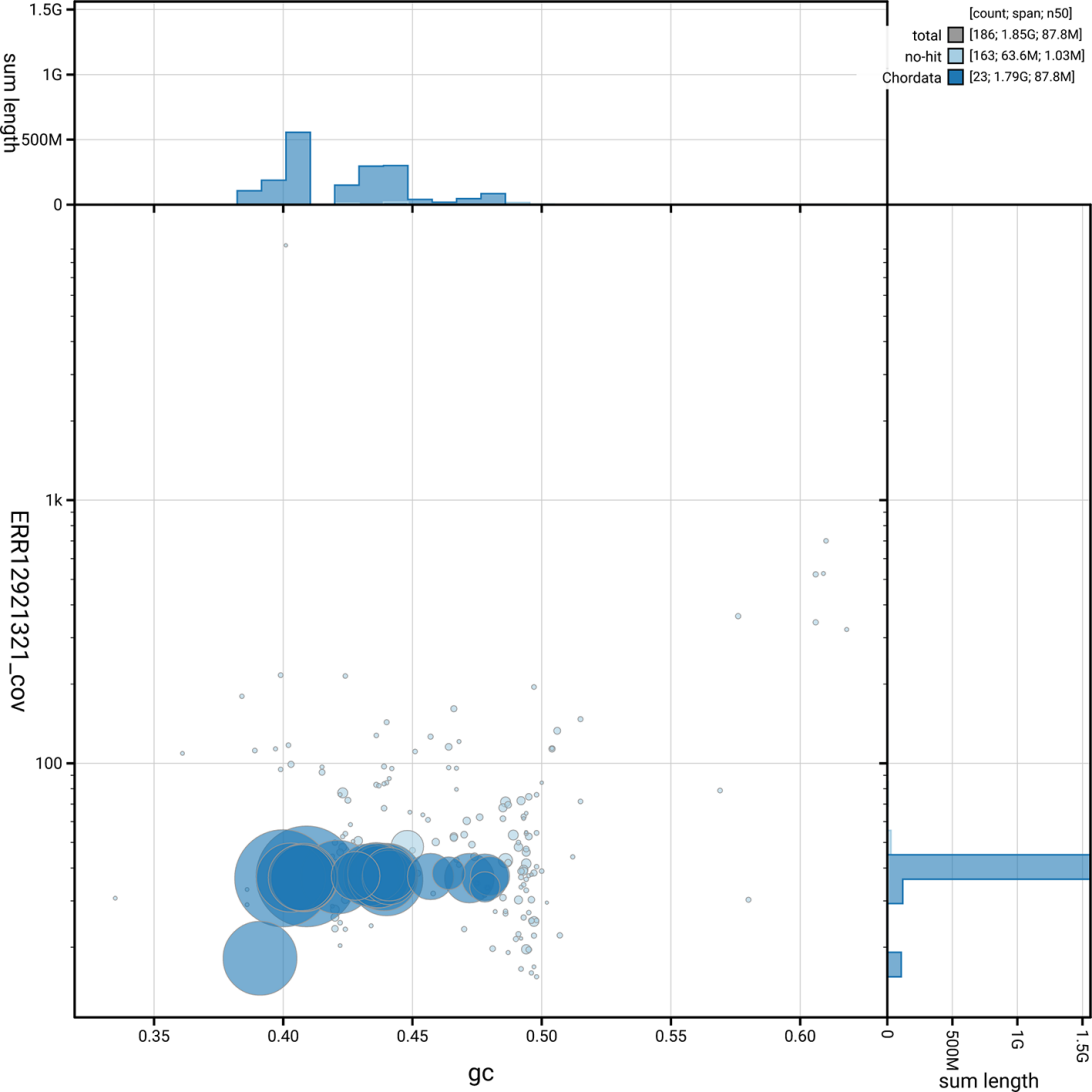
BlobToolKit blob plot for mMinSch1.hap1.2. The plot shows base coverage (vertical axis) and GC content (horizontal axis). The circles represent scaffolds, with the size proportional to scaffold length and the colour representing phylum membership. The histograms along the axes display the total length of sequences distributed across different levels of coverage and GC content. An interactive version of this figure is available on the BlobToolKit viewer.

BUSCO analysis using the metazoa_odb10 reference set ( *n* = 954) identified 99.9% of the expected gene set (single = 95.5%, duplicated = 4.4%) in haplotype 1. For haplotype 2, BUSCO v.6.0.0 analysis identified 98.5% of the expected gene set (single = 95.7%, duplicated = 2.8%). The snail plot in Figure 5 summarises the scaffold length distribution and other assembly statistics for haplotype 1. The blob plot in Figure 6 shows the distribution of scaffolds by GC proportion and coverage for haplotype 1.

Table 4 lists the assembly metric benchmarks adapted from Rhie *et al.* (2021) and the Earth BioGenome Project Report on Assembly Standards September 2024. The EBP metric, calculated for the haplotype 1, is **7.C.Q61**, meeting the recommended reference standard.

**Table 4.** Earth biogenome project summary metrics for the *Miniopterus schreibersii* assembly.

Measure	Value	Benchmark
EBP summary (haplotype 1)	7.C.Q61	6.C.Q40
Contig N50 length	28.01 Mb	≥ 1 Mb
Scaffold N50 length	87.80 Mb	= chromosome N50
Consensus quality (QV)	Haplotype 1: 61.8; haplotype 2: 61.7; combined: 61.8	≥ 40
*k*-mer completeness	Haplotype 1: 97.74%; Haplotype 2: 91.88%; combined: 99.79%	≥ 95%
BUSCO	C:99.9% [S:95.5%, D:4.4%], F:0.0%, M:0.1%, n:954	S > 90%; D < 5%
Percentage of assembly assigned to chromosomes	97.59%	≥ 90%

**Notes:** The EBP summary uses log10(Contig N50); chromosome-level (C) or log10(Scaffold N50); Q (Merqury QV). BUSCO: C = complete; S = single-copy; D = duplicated; F = fragmented; M = missing; n = orthologues

**Table 5.** Software versions and sources.

Software	Version	Source
BLAST	2.14.0	ftp://ftp.ncbi.nlm.nih.gov/blast/executables/blast+/
BlobToolKit	4.4.6	https://github.com/blobtoolkit/blobtoolkit
BUSCO	6.0.0	https://gitlab.com/ezlab/busco
bwa-mem2	2.2.1	https://github.com/bwa-mem2/bwa-mem2
DIAMOND	2.1.8	https://github.com/bbuchfink/diamond
fasta_windows	0.2.4	https://github.com/tolkit/fasta_windows
FastK	1.1	https://github.com/thegenemyers/FASTK
GenomeScope2.0	2.0.1	https://github.com/tbenavi1/genomescope2.0
Gfastats	1.3.6	https://github.com/vgl-hub/gfastats
Hifiasm	0.19.8-r603	https://github.com/chhylp123/hifiasm
HiGlass	1.13.4	https://github.com/higlass/higlass
MerquryFK	1.1.2	https://github.com/thegenemyers/MERQURY.FK
Minimap2	2.28-r1209	https://github.com/lh3/minimap2
MitoHiFi	3	https://github.com/marcelauliano/MitoHiFi
MultiQC	1.14; 1.17 and 1.18	https://github.com/MultiQC/MultiQC
Nextflow	24.10.4	https://github.com/nextflow-io/nextflow
PretextSnapshot	0.0.5	https://github.com/sanger-tol/PretextSnapshot
PretextView	1.0.3	https://github.com/sanger-tol/PretextView
samtools	1.21	https://github.com/samtools/samtools
sanger-tol/ascc	0.1.0	https://github.com/sanger-tol/ascc
sanger-tol/blobtoolkit	v0.9.0	https://github.com/sanger-tol/blobtoolkit
sanger-tol/curationpretext	1.4.2	https://github.com/sanger-tol/curationpretext
Seqtk	1.3	https://github.com/lh3/seqtk
Singularity	3.9.0	https://github.com/sylabs/singularity
TreeVal	1.4.0	https://github.com/sanger-tol/treeval
YaHS	1.2.2	https://github.com/c-zhou/yahs

### Genome annotation report

The *Miniopterus schreibersii* genome assembly (GCA_964146895.1) was annotated by Ensembl at the European Bioinformatics Institute (EBI). This annotation includes 43 915 transcribed mRNAs from 18 289 protein-coding and 7 391 non-coding genes. The average transcript length is 42 035.13 bp, with an average of 1.69 coding transcripts per gene and 9.66 exons per transcript. Further details of this annotation are available from the Ensembl annotation page.

## Author information

Contributors are listed at the following links:

• Members of the Wellcome Sanger Institute Tree of Life Management, Samples and Laboratory team
• Members of Wellcome Sanger Institute Scientific Operations – Sequencing Operations
• Members of the Wellcome Sanger Institute Tree of Life Core Informatics team
• Members of the Tree of Life Core Informatics collective
• Members of the Darwin Tree of Life Consortium

## Wellcome sanger institute – legal and governance

The materials that have contributed to this genome note have been supplied by a Darwin Tree of Life Partner. The submission of materials by a Darwin Tree of Life Partner is subject to the **‘Darwin Tree of Life Project Sampling Code of Practice’**, which can be found in full on the Darwin Tree of Life website. By agreeing with and signing up to the Sampling Code of Practice, the Darwin Tree of Life Partner agrees they will meet the legal and ethical requirements and standards set out within this document in respect of all samples acquired for, and supplied to, the Darwin Tree of Life Project. Further, the Wellcome Sanger Institute employs a process whereby due diligence is carried out proportionate to the nature of the materials themselves, and the circumstances under which they have been/are to be collected and provided for use. The purpose of this is to address and mitigate any potential legal and/or ethical implications of receipt and use of the materials as part of the research project, and to ensure that in doing so we align with best practice wherever possible. The overarching areas of consideration are:

• Ethical review of provenance and sourcing of the material
• Legality of collection, transfer and use (national and international)

Each transfer of samples is further undertaken according to a Research Collaboration Agreement or Material Transfer Agreement entered into by the Darwin Tree of Life Partner, Genome Research Limited (operating as the Wellcome Sanger Institute), and in some circumstances, other Darwin Tree of Life collaborators.

## Data Availability

European Nucleotide Archive: Miniopterus schreibersii (Schreibers’ long-fingered bat). Accession number PRJEB74978. The genome sequence is released openly for reuse. The *Miniopterus schreibersii* genome sequencing initiative is part of the Darwin Tree of Life Project (PRJEB40665), the Sanger Institute Tree of Life Programme (PRJEB43745), the Vertebrate Genomes Project (PRJNA489243) and the Bat1K project (PRJNA489245). All raw sequence data and the assembly have been deposited in INSDC databases. Raw data and assembly accession identifiers are reported in Tables 1 and 2. Production code used in genome assembly at the WSI Tree of Life is available at https://github.com/sanger-tol . Table 5 lists software versions used in this study.
