# Supplementary material for: The genome sequence of Schreibers’s Long-fingered Bat, Miniopterus schreibersii (Kuhl, 1817) (Chiroptera: Miniopteridae)
Source: Wellcome Open Res. 2026 Apr 22;11:233. [Version 1] doi: 10.12688/wellcomeopenres.26286.1 (PMC13197751; doi:10.12688/wellcomeopenres.26286.1)
Supplement: Peer review history

# Reviewer report: Reviewer response for version 1

**DOI:** 10.21956/wellcomeopenres.28948.r155046

**Published:** 2026-05-22

**Authors:** Edgar G Gutierrez

**Affiliations:** 1 National Polytechnic Institute, México, Santo Tomás, Mexico

The manuscript presents a valuable contribution to genomic resources, specifically providing a high-quality, chromosome-level genome assembly for the bat species *Miniopterus schreibersii*. The genomic data and assemblies presented are robust and will undoubtedly serve as an important tool for future research.

 The manuscript is generally well-structured and covers the methodological steps thoroughly. However, there are areas where the narrative flow, readability, and consistency could be significantly improved. In particular, some sections within the Background and Methods would benefit from a more logical restructuring to guide the reader more naturally from general concepts to specific findings. Additionally, there are minor technical inconsistencies between the text and figures (such as the colony size description and the BUSCO datasets) and a few formatting discrepancies in the software citations and references that need to be addressed. Restructuring these sections and addressing these minor revisions will greatly enhance the clarity, precision, and reproducibility of the study. I hope the following specific comments help the authors refine their manuscript.

 C: Unless the journal guidelines suggest otherwise, it is recommended to choose keywords that do not already appear in the title. This helps expand the article's discoverability and reach.

 C: The background section is informative, but the organization of ideas could be improved for better flow. In particular, the discussion of cryptic species and identification challenges appears before the manuscript introduces the taxonomic complexity of the genus *Miniopterus*. I suggest restructuring the paragraph so that the text progresses from general information about the species toward the more specific discussion of cryptic diversification and taxonomic reclassification. This may improve readability and narrative coherence.

 C: I suggest reorganizing the last background paragraph for a more natural narrative flow. Rather than beginning with the mention of a previously available scaffold-level assembly, the paragraph could first introduce the chromosome-level genome generated in this study and the sampled specimen, followed by contextual information about prior assemblies and related sequencing initiatives.

 C: The first Methods paragraph some sentences could be refined for smoother readability and more precise scientific wording. In particular, the sentence “One male individual was collected from these groups” appears somewhat redundant, as the paragraph already states that the sequenced specimen was an adult male *Miniopterus schreibersii*.

 C: In the Nucleic acid extraction paragraph, the repeated descriptions of nucleic acid quantification methods for both DNA and RNA could potentially be consolidated. Additionally, the introductory sentence regarding the availability of protocols may be integrated more smoothly into the paragraph for better narrative flow.

 C: The statement “Groups of *M. schreibersii* consisting of 2 to 10 individuals were observed roosting…” appears somewhat inconsistent with Figure 1, where a noticeably larger aggregation seems to be visible. The authors may wish to clarify whether the reported number refers to specific subgroups within the roost rather than the entire observed colony, in order to avoid potential confusion between the text and the figure.

 C: Unless the journal guidelines state otherwise, once a program is cited, it is no longer necessary to cite it each time it is mentioned in the text (e.g., the MerquryFK tool (Rhie et al., 2020); Singularity (Kurtzer et al., 2017), etc.).

 C: Could you please clarify which results table is being referenced here?: "The analysis outputs included assembly QV scores and completeness statistics."

 C: Chromosome 3 appears to show a distinct Hi-C interaction pattern relative to the other chromosomes. Although this may represent normal chromosomal organization, a brief explanation or comment in the manuscript would help readers interpret this feature and rule out potential assembly artefacts.

 C: In the sequence data paragraph, several sentences are repetitive and somewhat fragmented, particularly the repeated use of “which were used.” Consider reorganizing the sequencing and assembly information into a more concise structure, while clearly separating PacBio, Hi-C, and RNA-seq results. Additionally, the sentence “These estimates guided expectations for the assembly” is vague and could either be clarified or removed.

 Minor stylistic revisions would substantially improve clarity and readability. For instance, it is not entirely clear how to visualize the repetitive content mentioned in the text ("repeat content of 9.47% (Figure 2)") within Figure 2 itself.

 C: Consider defining the abbreviation “Mb” (megabases) at its first appearance, as was done for “Gb” (gigabases). Additionally, please standardize the capitalization of these units throughout the text; for example, "kb" is currently used in lowercase.

 C: In Table 5, consider removing the "v" from "sanger-tol/blobtoolkit v0.9.0" to maintain a standardized format in the version column across all listed software.

 C: The BUSCO software version should be reported in the Methods section, ideally at the first mention of the analysis, rather than appearing only in the second reference to BUSCO within the Results section. The current wording is somewhat inconsistent and may suggest that different BUSCO versions were used for each haplotype.

 C: Please check the BUSCO lineage dataset mentioned in the manuscript. The Results section indicates *metazoa_odb10*, while Figure 2 shows *laurasiatheria_odb10*; consequently, the values do not match. This inconsistency should be corrected for clarity and reproducibility.

 C: The species name *Miniopterus schreibersii* should be italicized throughout the manuscript, including in the Data Availability section.

 C: In the following references:

- Miller-Butterworth CM, Murphy WJ, O’Brien SJ, et al.:
- Ranallo-Benavidez TR, Jaron KS, Schatz MC:

The reference formatting appears inconsistent. Please abbreviate “Molecular Biology and Evolution” in accordance with the style used throughout the bibliography, and verify the punctuation and formatting (e.g., bold text) within the complete article title.

 C: Regarding the reference for Vasimuddin M, Misra S, Li H, et al., please check the page number formatting, as it appears inconsistent with the rest of the bibliography.

Are sufficient details of methods and materials provided to allow replication by others?

Yes

Is the rationale for creating the dataset(s) clearly described?

Yes

Are the datasets clearly presented in a useable and accessible format?

Yes

Are the protocols appropriate and is the work technically sound?

Yes

Reviewer Expertise:

immunogenetics, evolution, genomics, bioinformatics

I confirm that I have read this submission and believe that I have an appropriate level of expertise to confirm that it is of an acceptable scientific standard.

# Reviewer report: Reviewer response for version 1

**DOI:** 10.21956/wellcomeopenres.28948.r155043

**Published:** 2026-05-18

**Authors:** Ricardo Durães-Carvalho, Mariana Dias Guilardi

**Affiliations:** 1 Federal University of São Paulo, São Paulo, Brazil; 2 Institute of Chemistry, University of Sao Paulo, São Paulo, São Paulo, Brazil

**Review report n. 1**

**Summary**

 The authors present a high-quality chromosome-level genome assembly for the Schreibers’s long-fingered bat, *Miniopterus schreibersii* , generated from a male individual collected in Cyprus. The primary assembly (haplotype 1) spans 1.85 Gb and is scaffolded into 24 chromosomal pseudomolecules, including the X and Y chromosomes, with 97.59% of the sequence assigned to chromosomes. A secondary haplotype assembly (1.70 Gb) is also provided at scaffold level. In addition, the authors assembled the mitochondrial genome (16.63 kb) and report Ensembl annotation identifying 18,289 protein-coding genes.

 The assembly is of excellent quality, with a consensus QV of 61.8, BUSCO completeness of 99.9%, and combined k-mer completeness of 99.79%, surpassing current Earth BioGenome Project benchmarks. This work was produced as part of the Darwin Tree of Life and Bat1K initiatives and constitutes a valuable genomic resource for studies of bat evolution, conservation, comparative genomics, and host-virus interactions.

**Strengths**

**Technically robust approach.** The study uses state-of-the-art sequencing technologies, including PacBio HiFi long reads, Hi-C scaffolding, and RNA-seq support. Assembly and curation were performed using well-established tools such as Hifiasm, YaHS, MerquryFK, and BlobToolKit.

**Excellent assembly quality.** The resulting assembly exceeds Earth BioGenome Project standards (7.C.Q61), with high contiguity (contig N50 of 28.01 Mb), chromosomal completeness, and outstanding consensus accuracy.

**Comprehensive methodological description.** The manuscript provides sufficient details regarding sample collection, nucleic acid extraction, library preparation, sequencing, assembly, and quality assessment to enable reproducibility.

**Open and accessible data.** All raw sequencing data, assemblies, and associated metadata are publicly available through the European Nucleotide Archive under BioProject PRJEB74978.

**Clear biological relevance.** The background section effectively highlights the importance of this species for conservation genetics, phylogeography, and zoonotic disease research.

**Minor** **concerns a** **nd** **s** **uggestions**

 The manuscript is suitable for indexing in its current form. The following minor points may improve clarity and consistency:

**Species name consistency.** There are a few instances where the species epithet appears as *schreiberisii* rather than the correct *schreibersii* . The authors should verify and correct these typographical errors throughout the manuscript.

**Role of haplotype 2.** Although the second haplotype assembly is provided, its intended use is not discussed. A brief statement clarifying whether this assembly is included primarily for phasing and heterozygosity analyses, rather than for annotation, would be helpful.

**Single-individual reference.** As expected for a reference genome, the assembly is based on one specimen. It would be useful to acknowledge that this resource does not capture population-level genetic variation.

**Euthanasia details.** The legal and governance framework is clearly described, but the specific euthanasia method is not indicated. Including this information, or stating that it followed approved institutional protocols, would improve methodological transparency.

**Annotation accession version.** The annotation section cites accession GCA_964146895.1, whereas Table 2 lists GCA_964146895.2. The authors should confirm that the annotation corresponds to the latest assembly version.

****Aspects** **not assessed****

 I did not independently download or reanalyze the raw sequencing data, genome assembly, or annotation files. My evaluation is based on the information presented in the manuscript and the reported quality metrics.

 _____

**Review report n. 2**

 The authors provide a high-quality genome assembly of *Miniopterus schreibersii,* comprising two haplotypes at chromosome and scaffold-levels, respectively. This work presents an accurate description of the methods, including PacBIO HiFi and Hi-C and RNA-Seq, and the results of the assemblies. Results include haplotype-resolved assemblies, genome annotation, mitochondrial genome assembly, and quality evaluation, following the standards for vertebrate reference genomes. Some suggestions to improve the text are listed as follows:

 - Since external morphological features are insufficient for field identification, it would be important to include if wing, cranial or genetic data were used to identify the species prior NGS sequencing

 - If the specimen is deposited in a museum collection, the Methods could include its voucher number

 - The authors should include in the Background the information about cytogenetic data, considering that the assembly resulted in 24 chromosomal pseudomolecules, and the diploid number for the species is 2n=46 with polymorphisms in the autosomes (Aulagnier and Presetnik, 2020).

Are sufficient details of methods and materials provided to allow replication by others?

Yes

Is the rationale for creating the dataset(s) clearly described?

Yes

Are the datasets clearly presented in a useable and accessible format?

Yes

Are the protocols appropriate and is the work technically sound?

Yes

Reviewer Expertise:

Bioinformatics; Phylogeny and Molecular evolution; Genome assembly; Virology;  Cytogenetics; Small mammals.

We confirm that we have read this submission and believe that we have an appropriate level of expertise to confirm that it is of an acceptable scientific standard.

## References

[1] : Schreibers’ Bent-Winged Bat Miniopterus schreibersii (Kuhl, 1817). 10.1007/978-3-319-65038-8_48-1 1-26 10.1007/978-3-319-65038-8_48-1
